# Infant milk formulas differ regarding their allergenic activity and induction of T‐cell and cytokine responses

**DOI:** 10.1111/all.12992

**Published:** 2016-09-18

**Authors:** H. Hochwallner, U. Schulmeister, I. Swoboda, M. Focke‐Tejkl, R. Reininger, V. Civaj, R. Campana, J. Thalhamer, S. Scheiblhofer, N. Balic, F. Horak, M. Ollert, N. G. Papadopoulos, S. Quirce, Z. Szepfalusi, U. Herz, E. A. F. van Tol, S. Spitzauer, R. Valenta

**Affiliations:** ^1^ Division of ImmunopathologyDepartment of Pathophysiology and Allergy ResearchMedical University of ViennaViennaAustria; ^2^ Department of Medical & Chemical Laboratory DiagnosticsMedical University of ViennaViennaAustria; ^3^ Department of Molecular BiologyChristian Doppler Laboratory for Allergy Diagnosis & TherapyUniversity of SalzburgSalzburgAustria; ^4^ Allergy Centre Vienna WestViennaAustria; ^5^ Department of Infection and ImmunityLuxembourg Institute of Health (LIH)Esch‐sur‐Alzette, LuxembourgGermany; ^6^ Department of Dermatology and Allergy CenterOdense Research Center for AnaphylaxisUniversity of Southern DenmarkOdenseDenmark; ^7^ Allergy Research Center2nd Pediatric ClinicUniversity of AthensAthensGreece; ^8^ Center for Pediatrics and Child HealthInstitute of Human DevelopmentUniversity of ManchesterManchesterUK; ^9^ Department of AllergyHospital La Paz Institute for Health Research (IdiPAZ)MadridSpain; ^10^ Department of PediatricsMedical University of ViennaViennaAustria; ^11^ Mead Johnson NutritionEvansvilleINUSA; ^12^ Present address: Molecular Biotechnology SectionUniversity of Applied SciencesCampus Vienna BiocenterViennaAustria

**Keywords:** cow's milk allergy, hydrolyzed milk formulas, pro‐inflammatory cytokines, T‐cell reactivity

## Abstract

**Background:**

Several hydrolyzed cow's milk (CM) formulas are available for avoidance of allergic reactions in CM‐allergic children and for prevention of allergy development in high‐risk infants. Our aim was to compare CM formulas regarding the presence of immunoreactive CM components, IgE reactivity, allergenic activity, ability to induce T‐cell proliferation, and cytokine secretion.

**Methods:**

A blinded analysis of eight CM formulas, one nonhydrolyzed, two partially hydrolyzed (PH), four extensively hydrolyzed (EH), and one amino acid formula, using biochemical techniques and specific antibody probes was conducted. IgE reactivity and allergenic activity of the formulas were tested with sera from CM‐allergic patients (*n* = 26) in RAST‐based assays and with rat basophils transfected with the human FcεRI, respectively. The induction of T‐cell proliferation and the secretion of cytokines in Peripheral blood mononuclear cell (PBMC) culture from CM allergic patients and nonallergic individuals were assessed.

**Results:**

Immune‐reactive α‐lactalbumin and β‐lactoglobulin were found in the two PH formulas and casein components in one of the EH formulas. One PH formula and the EH formula containing casein components showed remaining IgE reactivity, whereas the other hydrolyzed formulas lacked IgE reactivity. Only two EH formulas and the amino acid formula did not induce T‐cell proliferation and proinflammatory cytokine release. The remaining formulas varied regarding the induction of Th2, Th1, and proinflammatory cytokines.

**Conclusion:**

Our results show that certain CM formulas without allergenic and low proinflammatory properties can be identified and they may also explain different outcomes obtained in clinical studies using CM formulas.

**Abbreviations**
CM: cow's milk
IgE: immunoglobulin E
RBL: rat basophil leukemia
rlf: recombinant lactoferrin
rα‐la: recombinant alpha‐lactalbumin
rαS1‐cas: recombinant alphaS1‐casein
rαS2‐cas: recombinant alphaS2‐casein
rβ‐cas: recombinant beta‐casein
rβ‐lg: recombinant beta‐lactoglobulin
rκ‐cas: recombinant kappa‐casein

Food allergy is increasing and represents an important public health problem 1, 2. Cow's milk (CM) is one of the most important allergen sources particularly in children and can elicit severe life‐threatening reactions in sensitized patients 3, 4. The molecular nature of CM allergens and the allergic immune responses in terms of antibody and cellular responses are subject of several studies with the goal to develop diagnostic, therapeutic, and preventive strategies for CM allergy 5.

For children who cannot be breastfed, the use of hydrolyzed CM formulas has been recommended for the prevention of allergic reactions to CM in allergic children (i.e. for treatment) as well as for the prevention of allergic sensitization and allergy development in high‐risk children 6, 7, 8, 9.

Cow's milk formulas differ regarding the degree of hydrolysis of the milk proteins as well as regarding the hydrolysis procedure ranging from partially to extensively hydrolyzed (EH) formulas. Amino acid substitutes are available for highly CM‐allergic infants. Furthermore, CM formulas are named depending on their protein source, such as whey or casein hydrolyzates 10. Partially hydrolyzed (PH) formulas are supposed to contain small and larger oligopeptides with a molecular weight of <5 kDa, EH formulas should contain only peptides with a molecular weight of <3 kDa, and amino acid‐based formulas (AA) are made of essential and nonessential amino acids 10.

In a series of early intervention studies using hydrolyzed CM formulas, it could be shown that certain formulas were useful for allergy prevention in the first year of life 11 and reduced the incidence of atopic dermatitis (AD) at the age of 3 and 6 years and this preventive effect persisted until the age of 10 years without rebound 12, 13, 14.

Here, we conducted a blinded analysis of eight CM formulas: one nonhydrolyzed, two PH, four EH, and one amino acid formula, regarding their biochemical composition, the presence of antibody‐reactive CM allergens/allergen fragments, IgE reactivity, abilities to induce basophil activation, T‐cell proliferation, and secretion of a panel of different cytokines. Our study revealed major differences among the formulas regarding the presence of immunogenic allergens/allergen fragments, IgE reactivity, allergenic activity, induction of T‐cell responses, and cytokine secretion. In particular, we were able to demonstrate a strongly varying capacity of the formulas to induce the secretion of Th1, Th2, and other proinflammatory cytokines. Our results may provide not only an explanation for the selective effects of CM formulas on the prevention of allergic sensitization and certain allergic manifestations. They also indicate that CM formulas with low proinflammatory activity can be identified, which may have potential for prevention of other inflammatory diseases.

## Materials and methods

### Biological materials

In total, 10 coded CM formulas were analyzed in a blinded manner regarding their biochemical and immunological characteristics. Only after completion of the analysis, their identity was disclosed. Table 1 provides a summary and characterization of the 10 CM formulas regarding their manufacturer, source, degree of hydrolysis, protein, and endotoxin contents.

**Table 1:** Characterization of milk formulas M1–M10

Milk samples	Product	Manufacturer	Source (casein or whey)	Condition	Protein content (g/100 g)	Endotoxin content (EU in 100 μg protein)
M1	Enfamil premium	MJN	C + W	Nonhydrolyzed (NH)	11.00	0.035
M2	Enfamil HA‐Gentlease	MJN	C + W	Partially (PH)	12.80	0.035
M3	Nutramigen	MJN	C	Extensively (EH)	14.00	0.036
M4	Nutramigen AA	MJN	AA	Amino acids (AA)	14.00	0.041
M5	Nan HA	Nestle	W	Partially (PH)	11.50	0.034
M6	Friso allergycare	Friso	C	Extensively (EH)	11.70	0.036
M7	Alimentum advance	Ross	C	Extensively (EH)	13.93	0.030
M8	Alfare	Nestle	W	Extensively (EH)	14.80	0.039
M9	Milk protein	MJN	C + W	Whole milk whey and casein proteins	36.00	0.046
M10	Whey	MJN	W	Whole whey proteins	36.10	0.048

HA, hypoallergenic; MJN, Mead Johnson Nutrition; C, casein; W, whey; AA, amino acids; EH, extensively hydrolyzed; PH, partially hydrolyzed.

The endotoxin levels of the formulas were measured with Pierce LAL Chromogenic Endotoxin Quantitation Kit (Thermo Scientific, Vienna, Austria) as described in the user manual.

Antibody probes specific for the individual CM proteins were produced as follows: cDNA coding for several CM allergens were isolated by IgE immunoscreening of a cDNA expression library prepared from bovine mammary glands 15. Recombinant CM allergens (alphaS1‐casein, alphaS2‐casein, beta‐casein, kappa‐casein, alpha‐lactalbumin, beta‐lactoglobulin, lactoferrin) were expressed in *Escherichia coli* strain BL21 Codon Plus (DE3)‐RIPL (Stratagene, La Jolla, CA, USA) as hexahistidine‐tagged proteins and purified by Ni^2+^ affinity chromatography (QIAGEN, Hilden, Germany) as described by Schulmeister et al. 15.

Allergen‐specific rabbit antibodies were obtained by immunizing rabbits three times (once in CFA and twice in incomplete Freund's adjuvant (IFA)) with the purified recombinant proteins (Charles River, Kisslegg, Germany).

### Serum and blood samples

Serum and blood samples were obtained from CM‐allergic patients (*n* = 26), patients who suffered from symptoms after CM consumption but without CM‐specific IgE (*n* = 2), subjects with CM‐specific IgE but without symptoms (*n* = 4), and from six nonallergic subjects. The diagnosis of CM allergy was based on the presence of clinical symptoms that could be unambiguously attributed to consumption of CM and/or on results of an open food challenge, a positive skin prick test reaction, and the presence of specific IgE to CM allergens as measured by ImmunoCAP (Thermo Fisher Scientific, Uppsala, Sweden) (Table 2). Hypolactasia was not investigated in the CM‐allergic patients because it does not affect immune reactivity to CM components. In addition to the CM‐allergic subjects, we tested also serum and blood samples from nonallergic subjects, two patients with symptoms upon CM contact (cough, gastrointestinal problems) lacking CM‐specific IgE, and four subjects with CM‐specific IgE without symptoms to CM (Table 2). Serum and blood samples were analyzed in an anonymized manner with permission of the Ethics Committee of the Medical University of Vienna (EK565/2007; EK1641/2014). For freshly taken blood samples, informed written consent was obtained from the subjects. Clinical and demographic features of the subjects are summarized in Table 2.

**Table 2:** Demographic and clinical features of patients and control individuals

Patient	Sex M/F	Age	Milk‐related symptoms	Other allergies	Total IgE (kU/l)	Spec. IgE to CM (kUA/l)
A: Nonallergic individuals (n = 6)	2/4	21–51 years	No	No	5.8–91.1	<0.35
B: CM allergic patients (n = 26)	14/9 3 nk	4 months to 70 years	AD, AE, AS diarrhea, E, eczema GI, Rh, U, V, Sys	Animal dander, candida, cat, dog fish, hazelnut, HE, mite, moulds nuts, PO, soy, wheat	3.58–3350	1.3–>100
C: Patients without CM‐specific IgE but with symptoms (n = 2)	1/1	25–53 years	CO, GI	Cat, mite	64.9–153	<0.35
D: Patients with CM‐specific IgE but without symptoms (n = 4)	2/2	5–55 years	No	Birch, HE, PO, sheep milk	14.1–1844	0.79–7

F, female; M, male; Symptoms: AD, atopic dermatitis; AE, angioedema; AS, asthma; E, edema; GI, gastrointestinal symptoms; Rh, rhinitis; U, urticaria; V, vomiting; Sys, systemic reactions; CO, cough; Allergen (source): HE, hen's egg; PO, pollen; kU/l, total IgE in kilo units/liter; kUA/l, allergen‐specific IgE in kilo units antigen/liter; CM, cow's milk; nk, not known.

### Analysis of formulas by SDS‐PAGE and by dot blotting with specific antibody probes

Protein, peptide, and amino acid contents in the milk formulas were determined by measuring protein nitrogen in the samples by the Kjeldahl method 16. Aliquots of 30 μg/lane of the milk samples were subjected to SDS‐PAGE and Coomassie Brilliant Blue staining 17. For immunoblot analysis, 1 μg aliquots of the milk samples were dotted onto a nitrocellulose membrane (Schleicher & Schuell, Dassel, Germany). Dot blotting instead of Western blotting was chosen in order not to lose small peptides during gel electrophoresis and electroblotting and to avoid denaturing conditions that may affect IgE reactivity. The nitrocellulose strips were blocked with PBST (PBS, 0.5% v/v Tween 20) and exposed to rabbit antisera (1 : 2000 diluted) or to sera from CM‐allergic patients and nonallergic individuals (1 : 10 or 1 : 20 diluted) overnight at 4°C. Bound rabbit IgG antibodies were detected with ^125^I‐labeled donkey anti‐rabbit IgG (Perkin Elmer, Boston, MA, USA) diluted 1 : 2000 in PBST or in the case of human IgE antibodies with ^125^I‐labeled anti‐human IgE antibodies (IBL, Hamburg, Germany), diluted 1 : 15. Positive signals were visualized by autoradiography using Kodak XOMAT films with intensifying screens (Kodak, Vienna, Austria) at −80°C.

### Rat basophil leukemia assays

For the assessment of the allergenic activity of the milk samples, huRBL cell mediator release assays were performed as described previously 15, 18. In brief, rat basophil leukemia (RBL) cells (clone RBL‐703/21) transfected with the human FcεRI were incubated with sera from CM‐allergic patients overnight. On the next day, the cells were washed, and 100 μl of milk components (concentration: 0.3 μg/ml total protein contents) were added and incubated for 1 h at 37°C, 7% CO_2_, 95% humidity. Aliquots of the supernatants were mixed with assay solution (0.1 M citric acid or sodium citrate, pH4.5 and 160 μM 4‐methyl umbelliferyl‐*N*‐acetyl‐β‐d‐glucosamide) and incubated for 1 h at 37°C, 7% CO_2_, 95% humidity. Fluorescence was measured with a fluorescence microplate reader, and specific release could be calculated. Values obtained with buffer alone were subtracted, and the values exceeding 10% of total release were considered as positive.

### Lymphocyte proliferation assays

PBMCs from six nonallergic individuals and seven CM‐allergic patients were isolated from heparinized blood samples by Ficoll density gradient centrifugation (Amersham Biosciences, Uppsala, Sweden). PBMCs (2 × 10^5^ cells per well) were cultured in triplicates in 96‐well plates (Nunclone; Nalgen Nunc International, Roskilde, Denmark) in 200 μl serum‐free Ultra Culture medium (BioWhittaker, Rockland, ME, USA) supplemented with 2 mM l‐glutamine (Gibco, Carlsbad, CA, USA), 50 μM β‐mercaptoethanol (Gibco), and 0.1 mg gentamicin per 500 ml (Gibco). The cells were incubated at 37°C in a humidified atmosphere with 5% CO_2_ for 7 days and stimulated with different concentrations of milk samples (0.05, 0.5, 3, and 10 μg/well), 4 U IL‐2 per well (Roche) as a positive control and medium alone as a negative control in duplicate. After 6 days of incubation, 0.5 mCi ^3^H‐thymidine (Amersham, Buckinghamshire, UK) was added to each well for 16 h, and then, the incorporated radioactivity was measured by liquid scintillation counting. Proliferation was expressed as counts per minute (c.p.m.; means of triplicates) using a microbeta scintillation counter (Wallac ADL, Freiburg, Germany). The mean stimulation indices (SI) were calculated as quotient of triplicate c.p.m. with antigen *vs* medium and shown are the SI obtained by stimulation with 10 μg protein/well.

### Analysis of cytokine levels in supernatants

Cytokine levels (IL‐5, IL‐6, IL‐10, IL‐13, IFN‐γ, TNF‐α, GM‐CSF) were measured in supernatants collected from PBMC cultures at day 6 of culture using xMAP Luminex fluorescent bead‐based technology. The assays were performed according to the manufacturer's instructions (R&D Systems, Wiesbaden, Germany), and fluorescent signals were read on a Luminex 100 system (Luminex Corp., Austin, TX, USA). The limits of detection were 1.9 pg/ml for IL‐5, 5.5 pg/ml for IL‐6, 3.1 pg/ml for IL‐10, 47 pg/ml for IL‐13, 2.9 pg/ml for IFN‐γ, 5.3 pg/ml for TNF‐α, and 3.3 pg/ml for GM‐CSF. Shown are means of triplicate determinations from cultures stimulated with 10 μg protein/well.

### Statistics

Statistical comparisons were performed by Mann–Whitney *U*‐test for nonparametric values. *P*‐values < 0.05 were considered as significant. For all calculations, the statistical program pasw Statistics 18 (Version 18.0.0. 1993–2007; Polar Engineering and Consulting Nikiski, Alaska, United States) was used.

## Results

### Biochemical and immunochemical analysis indicates different compositions of milk formulas

**Figure 1:**
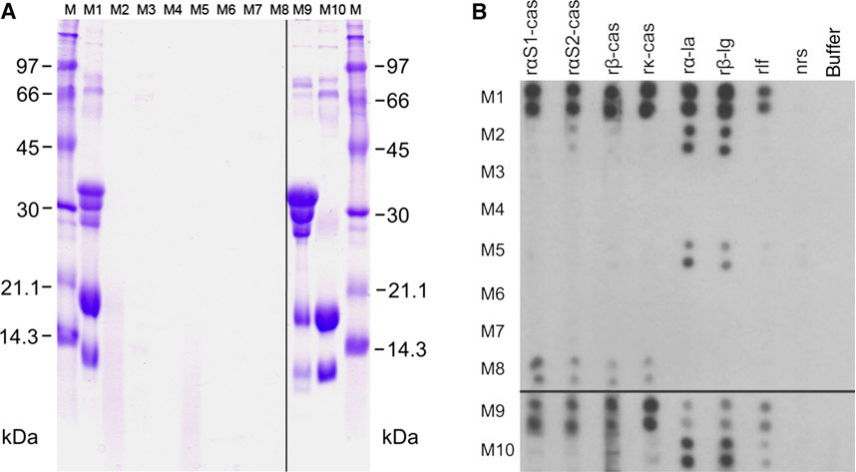
(A) Detection of proteins in milk formulas M1–M10 by SDS‐PAGE and Coomassie Brilliant Blue Staining. Aliquots of each milk formula (lanes M1–M10) were loaded. Lanes M show molecular weight markers (kDa). (B) Reactivity of M1–10 with specific antibody probes. Aliquots of the samples were dotted in duplicates onto nitrocellulose and incubated with rabbit antibodies raised against recombinant cow's milk (CM) proteins (rαS1‐cas, rαS2‐cas, rβ‐cas, rκ‐cas, rα‐la, rβ‐lg, rlf), with normal rabbit serum (nrs) or with buffer alone.

In the first step, formulas M1 to M10 were analyzed by SDS‐PAGE and stained with Coomassie Brilliant Blue to visualize intact proteins. This analysis demonstrated the presence of several proteins with molecular masses between 10 and 100 kDa in the formulas M1, M9, and M10 and to lesser extent in M3 (Fig. 1A). Protein smears below 20 kDa were found in M2 and M5, whereas in M4, M6, M7, and M8, no protein staining was observed (Fig. 1A). The measurement of the endotoxin levels in the 10 samples showed low levels of endotoxin (<0.05 EU in 100 μg protein) (Table 1).

In the next step, we used specific antisera raised against purified recombinant CM allergens (rαS1‐cas, rαS2‐cas, rβ‐cas, rk‐cas, rα‐la, rβ‐lg, rlf) to detect immunoreactive components in the formulas in dot blot experiments (Fig. 1B). The whole CM protein‐containing formula M1 and the milk protein control M9 reacted with each of the antisera demonstrating the presence of the proteins of the casein fraction (αS1‐cas, αS2‐cas, β‐cas, k‐cas) and from the whey fraction (α‐la, β‐lg, lf). In M2, a PH formula that is made up from casein and whey, mainly α‐la and β‐lg and to a much lower degree, αS2‐cas was detected. The other PH formula M5 also contained immunoreactive α‐la and β‐lg. Interestingly, the EH formula M8 that is produced of the whey fraction contains mainly the immunoreactive caseins but whey protein could not be detected. In the whey fraction M10, each of the whey proteins (i.e. α‐la, β‐lg, and lactoferrin) was detected. No immunoreactive proteins of the casein and whey fraction were detected in the formulas M3, M4, M6, and M7.

### Milk formulas show major differences regarding IgE reactivity and allergenic activity

**Figure 2:**
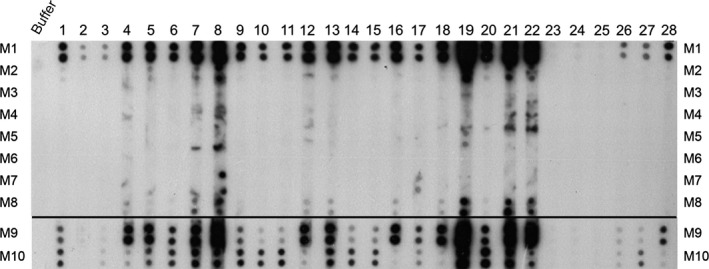
IgE reactivity of dot‐blotted milk samples M1–M10. Aliquots of the sample were dotted in duplicates onto nitrocellulose and incubated with buffer alone, with sera from cow's milk (CM)‐allergic patients 1, 3, 4, 5, 6, 7, 8, 9, 10, 11, 12, 13, 14, 15, 16, 17, 18, 19, 20, 21, 22, with sera from subjects who had problems after milk consumption but lacked CM‐specific IgE 23, 24, with serum from a nonallergic control 25, and with sensitized individuals who had CM‐specific IgE without symptoms 2, 26, 27, 28.

In the next step, the IgE reactivities of the samples were tested with a highly sensitive RAST‐based dot blot analysis using sera from 21 CM‐allergic patients, two patients with symptoms after CM consumption lacking milk‐specific IgE, a nonallergic control, and four patients with CM‐specific IgE antibodies but without symptoms (Fig. 2). Almost each of the 21 CM‐allergic patients showed IgE reactivity to M1, M9, and M10. There were differences regarding IgE reactivity to M1, M9, and M10, which may be attributed to a different sensitization of the patients to caseins and whey proteins. IgE reactivity to M8 was found for patients 7, 8, 12, 13, 16, 18, 19, 21, and 22 (Fig. 2). Patients 1, 5, 7, 8, 19, 20, 21, and 22 reacted with M2. Patient 19 and 17 showed weak IgE reactivity to M5 and M7, respectively (Fig. 2). Patients 8 and 17 showed reactivity to M7. None of the other formulas M3, M4, and M6 showed relevant IgE reactivity. The control serum from the nonallergic person 25 showed no IgE reactivity to any of the dotted formulas (Fig. 2).

The assessment of the allergenic activity of the milk formulas by basophil degranulation experiments was in quite good agreement with the IgE reactivity data. We found that mainly the samples M1, M9, and M10 induced mediator release in CM‐allergic patients. Furthermore, M8 induced degranulation in cells loaded with sera from patients 19 and 22 (data not shown).

### Different capacity of milk formulas to induce lymphocyte proliferation

**Figure 3:**
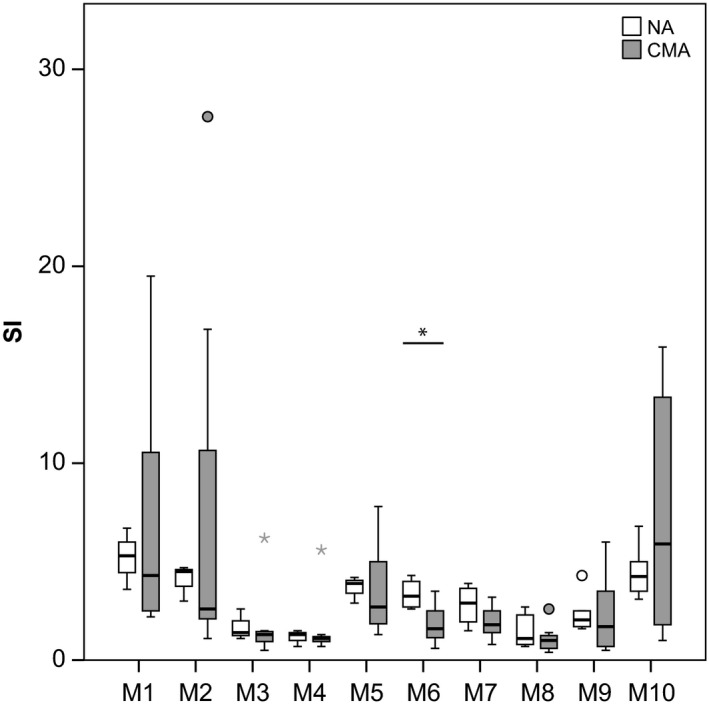
Lymphoproliferative responses in PBMCs induced by milk formulas M1–M10. PBMCs from six nonallergic individuals and from seven cow's milk (CM)‐allergic patients were stimulated with milk formulas (M1–M10) (*x*‐axis). Box plots of stimulation indices with indicated medians for nonallergic (white) and allergic subjects (gray) are displayed (*y*‐axis). Asterisks are extreme outliers, and circles represent mild outliers. *Statistical significant difference (*P* < 0.05).

Next, we tested the ability of the milk formulas to induce lymphocyte proliferation in cultured PBMCs from seven CM‐allergic and six nonallergic individuals (Fig. 3). Except for the whole milk preparation M9, nonhydrolyzed (M1, M10) and PH formulas (M2, M5) showed the highest median SI (M1: nonallergic: SI 5.3, CM allergic: SI 4.3; M2: nonallergic: SI 4.5, CM allergic: SI 2.6; M5: nonallergic: SI 3.9, CM allergic: SI 2.7, M9: nonallergic: SI 2.1, CM allergic: SI 1.8) (Fig. 3). Among the EH formulas, the median SI were higher for M6 (nonallergic: SI 3.3, CM allergic: SI 1.6) and M7 (nonallergic: SI 2.9, CM allergic: SI 1.8), whereas M3 (nonallergic: SI 1.4, CM allergic: SI 1.3) and M8 (nonallergic: SI 1.1, CM allergic: SI 1.0) revealed lowest proliferation. The proliferation induced with M3 and M8 was as low as that obtained for the amino acid formulation M4 (nonallergic: SI 1.3, CM allergic: SI 1.1). It was interesting to note that milk formulas containing immunoreactive whey proteins (Fig. 1B: M1, M2, M5, M9, and M10) showed higher lymphocyte proliferation than the milk formula containing only caseins (Fig. 1B: M8). There were no statistical significant differences between the median SI observed for milk allergic and nonallergic individuals except for M6 with nonallergic individuals showing significantly higher SI (Fig. 3, *P* < 0.024).

### Identification of milk formulas that induce low levels of proinflammatory cytokines

**Figure 4:**
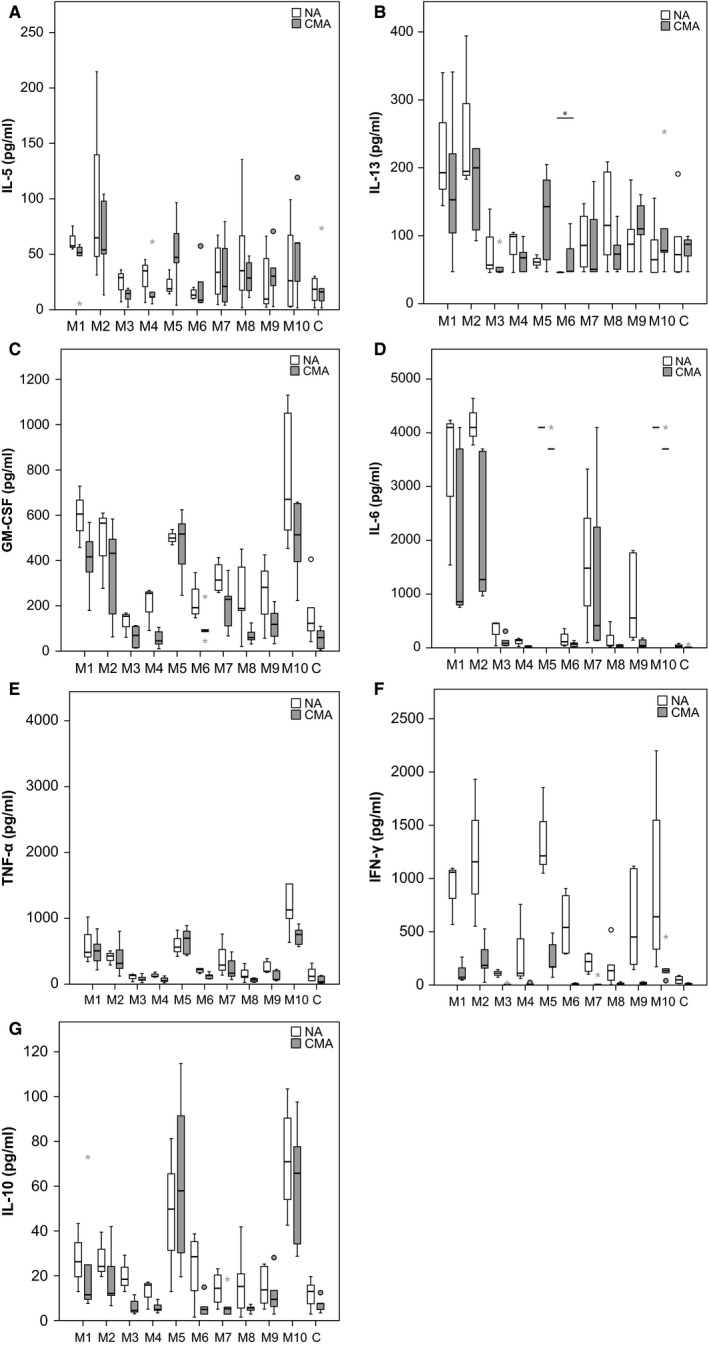
Cytokine responses in PBMCs induced by milk formulas M1–M10. PBMCs from six nonallergic individuals (NA) and from five cow's milk (CM)‐allergic patients were stimulated with milk formulas (M1–M10) or medium control (C). Shown are box plots of cytokine levels with indicated medians for nonallergic (white) and allergic subjects (gray) (*y*‐axis). Asterisks are extreme outliers, and circles represent mild outliers. Ranges of detection (*y*‐axes) for IL‐5 (A): 1.9–1400 pg/ml, for IL‐13 (B): 47–34 500 pg/ml, for GM‐CSF (C): 3.3–2400 pg/ml, for IL‐6 (D): 5.5–4000 pg/ml, for TNF‐α (E): 5.3–3900 pg/ml, for IFN‐γ (F): 2.9–2100 pg/ml and IL‐10 (G): 3.3–2225 pg/ml.

The PBMC culture supernatants from nonallergic individuals and CM‐allergic patients stimulated with the milk formulas M1–M10 were analyzed regarding the secretion of various cytokines by Luminex analysis (Fig. 4). The EH formulas M3 and M6 and the amino acid formulation M4 were the milk formulas that induced low levels of all tested cytokines. Similarly, the EH formula M8 induces low levels for most cytokines except for IL‐5.

When taking a closer look at the Th2 cytokines such as IL‐5 (Fig. 4A) and IL‐13 (Fig. 4B), the following observations were made: First, all but M6 induced IL‐13. There were no significant differences between CM allergic and nonallergic subjects regarding the levels of IL‐5 and IL‐13 in the stimulated cultures (Fig. 4A,B). Second, M2, a PH formula, induced IL‐5 and IL‐13 as strongly as the nonhydrolyzed formula M1, whereas the other formulas were less active (Fig. 4A,B).

Similar findings were made for GM‐CSF. Again, M1 and M2 induced high levels of GM‐CSF, but in this case, also the other PH formula M5, and M10, the whole whey proteins, induced high levels of GM‐CSF (Fig. 4C). Another difference as compared to IL‐5 and IL‐13 was that for all formulas but M5, GM‐CSF levels were lower in PBMC cultures of allergic patients, although this was not statistically significant.

Next, we analyzed the proinflammatory cytokines IL‐6 and TNF‐α that gave similar profiles for the different formulas. The highest levels of IL‐6 and TNF‐α were found for formulas containing complete proteins (i.e. M1, M10), and the PH formulas M2 and M5 and, interestingly, also the EH formula M7 induced high levels of these cytokines (Fig. 4D,E). There was however one difference: IL‐6 levels were higher in PBMC cultures of nonallergic subjects than in cultures of allergic patients, whereas no such differences were noted for TNF‐α (Fig. 4D,E).

Regarding the Th1 cytokine IFN‐γ, we found that IFN‐γ levels were always higher in PBMC cultures from nonallergic subjects (Fig. 4F). Again, formulas containing complete proteins (M1, M10) but also the PH formulas M2 and M5 induced the highest levels of IFN‐γ, followed by the EH formulas M6, M7, and the whole milk sample M9 (Fig. 4F).

For the IL‐10, different profiles were observed: The highest levels of IL‐10 were induced by the PH formula M5 and the complete whey proteins M10 (Fig. 4G). For M5 and M10, we did not find significant differences between allergic and nonallergic subjects. By contrast, IL‐10 levels were always lower for each of the other tested formulas in allergic subjects.

In summary, the EH formulas M3 and M6 as well as the amino acid formulation M4 were the formulas which induced the least allergic/proinflammatory cytokine production in PBMCs from allergic as well as nonallergic subjects.

## Discussion

Hydrolyzed CM formulas are used widely in the diet of CM‐allergic children to prevent allergic reactions and for the prevention of allergic sensitization and allergy development in high‐risk children. Here, we performed a blinded analysis of 10 different CM formulas regarding the presence of immune‐reactive CM allergens/allergen fragments, IgE reactivity, allergenic activity, ability to stimulate T‐cell responses, and the secretion of a panel of different cytokines. In fact, hydrolyzed CM formulas have been tested already earlier regarding IgE reactivity, allergenic activity, and *in vivo* allergenicity 19, 20, but the results of our study revealed some additional interesting aspects. It is assumed that extensively hydrolyzed CM formulas are less IgE‐reactive and allergenic 10. Furthermore, it has been shown that EH formulas exhibit lower antigenicity and allergenicity when fed to infants than PH CM formulas 21. However, our analysis demonstrates that certain EH formulas (e.g. M7, M8) similar as PH formulas (M2, M5) exhibited remaining IgE reactivity and/or allergenic activity in basophil activation assays. Another interesting observation was that with the use of antibody probes specific for certain CM allergens, immune‐reactive casein fragments were detected in formula M8 that was prepared from whey. It is thus quite likely that the residual IgE reactivity and allergenic activity of this formula was due to the presence of remaining allergenic casein‐derived material. In fact, a similar finding was made recently by authors who studied time courses of whey hydrolysis and actually found that even after prolonged hydrolysis, patients showed IgE reactivity to caseins in the preparations 22. It is thus possible that hydrolyzed whey preparations may contain casein‐derived peptides because they are less well hydrolyzed than the whey components.

The analysis of the capacity of the formulas to induce T‐cell proliferation and cytokine secretion showed further differences among the formulas. The results of the T‐cell proliferation experiments yielded similar results as observed earlier, in that PH formulas induced T‐cell proliferation almost to the same extent as formulas containing complete allergen 23. However, only two of the four EH formulas (M3, M4) showed basically no remaining T‐cell reactivity such as the amino acid formulation and thus proved to be non‐T‐cell stimulatory. Cow's milk formulas in which immune‐reactive whey proteins were detected (i.e. M1, M2, M5, and M10) induced higher lymphocyte proliferation than the formula containing only caseins (i.e. M8). Interestingly, there were no relevant differences regarding the induction of T‐cell proliferation between CM‐allergic and nonallergic individuals. As the CM formulas are natural products, it cannot be excluded that carbohydrates or lipids in the formulas had an influence on the cellular responses but it is unlikely because proteins/peptides represent the major constituents of the CM formulas and the endotoxin levels in the preparations were very low.

The perhaps most interesting results came from the analysis of the induction of cytokines upon stimulation of PBMC with the CM formulas. In fact, we noted significant differences of the CM formulas to induce Th2, Th1, and proinflammatory cytokine responses. There were also significant differences regarding the production of proinflammatory cytokines such as IL‐6 and the Th1 cytokine IFN‐γ between allergic and nonallergic individuals. PBMC from nonallergic individuals secreted higher levels of IL‐6 and IFN‐γ than PBMCs from allergic individuals. It is possible that the proinflammatory cytokines are not exclusively derived from T cells, but this will reflect *in vivo* conditions where also other cell types are present similar as in PBMCs.

Most importantly, we found that three formulas (EH: M3 and M6; amino acid formulation: M4) did not induce any relevant levels of Th1, Th2, or proinflammatory cytokines, neither in PBMCs of allergic or nonallergic patients. This finding can most likely be explained by the lack of immune‐stimulatory peptides in these formulas which is consistent with the observation that they also did not stimulate T‐cell proliferation. Interestingly, the formula M3 had been shown in the long‐term German Infant Nutritional Intervention Study (GINI) to reduce the risk of developing AD, a T‐cell‐driven allergic skin manifestation 11. However, it must be noted that it is presently not clear if the induction of proinflammatory cytokines in PBMC samples is related to clinical symptoms.

In summary, our results demonstrate that there are striking differences among hydrolyzed CM formulas regarding IgE reactivity, allergenic activity, and the ability to induce proinflammatory immune responses. It is quite possible that some of the discrepancies observed in clinical intervention trials and in the course of the clinical use of CM formulas for the prevention and treatment of CM allergy could be due to differences in the immunological and/or immunomodulatory properties of the various available preparations. It would thus seem to make sense to agree on common standardization protocols for the various CM formulas 24 similar as are used for example for standardization of diagnostic and therapeutic allergen extracts 25.

The finding that it is possible to identify CM formulas without allergenic activity and no proinflammatory activity makes it tempting to speculate that such formulas might be useful not only for the prevention of CM allergy but maybe also for other inflammatory diseases. In this context, it has been suggested that inflammation in the gut and inflammatory processes leading to defects in the mucosal gut barrier may contribute to local and systemic autoimmunity 26, 27, 28, 29, 30.

## Author contributions

HH and RC performed experiments, analyzed data, wrote manuscript, and read manuscript; USCH, IS, MFT, SS, NB, and RR performed experiments, analyzed data, and read manuscript; JT, FH, MO, NGP, SQ, ZS, UH, EVT, and SSP analyzed data and read manuscript; and RV designed and supervised experiments, analyzed data, wrote manuscript, and read manuscript.

## Conflicts of interest

Rudolf Valenta and Heidrun Hochwallner have received research grants from the Austrian Science Fund (FWF). Rudolf Valenta has received research grants from Biomay AG, Vienna, Austria, and Thermofisher, Uppsala, Sweden, and serves as a consultant for these companies.
